# Significance of glomerular fibrinogen deposition in children with Henoch-Schönlein purpura nephritis

**DOI:** 10.1186/s13052-018-0538-1

**Published:** 2018-08-16

**Authors:** Fengying Wang, Lusheng Huang, Hangyun Tang, Xiaozhong Li, Xueming Zhu, Xingdong Wang

**Affiliations:** 1 Department of Pediatrics, Taixing Hospital Affiliated to Yangzhou University, Taixing, Jiangsu China; 2 Department of Nephrology and Immunology, Soochow University Affiliated Children’s Hospital, Suzhou, Jiangsu China; 3 Department of Pathology, Soochow University Affiliated Children’s Hospital, Suzhou, Jiangsu China

**Keywords:** Fibrinogen deposition, Henoch-Schönlein purpura nephritis, Children

## Abstract

**Background:**

Henoch-Schönlein purpura nephritis (HSPN) is the most common pediatric secondary glomerular disease. This study aimed to investigate the significance of glomerular fibrinogen (Fib) deposition in children with HSPN.

**Methods:**

Eighty-two patients with HSPN were enrolled retrospectively at the Children’s Hospital of Soochow University from January 2015 to March 2017. Patients were divided into groups according to the presence or absence and intensity of glomerular Fib deposits, and clinical and pathological features were compared among the groups.

**Results:**

Glomerular Fib deposition was observed in 64 children (78.05%), including 1 Fib± case (1.22%), 23 Fib+ cases (28.05%), 37 Fib++ cases (45.12%), and 3 Fib+++ cases (3.66%). Significantly different levels of high-sensitivity C-reactive protein (hs-CRP), D-dimer (DD), proportions of CD19 + CD23+ cells and urine microalbumin:creatinine ratios (UMA/Cr) were noted among the different Fib deposition groups (no, mild and severe). Pairwise comparison in multiple groups revealed significantly increased hs-CRP, proportion of CD19 + CD23+ cells and UMA/Cr in the severe deposition group compared with the mild and no deposition groups, and remarkably increased DD levels were noted in the severe and mild deposition groups compared with the no deposition group. The degree of glomerular Fib deposition was positively correlated with the degree of glomerular IgA deposition, and the incidence of glomerular IgG deposition in the severe deposition group was increased compared with the no deposition group.

**Conclusion:**

HSPN children with glomerular Fib deposition, especially those with severe Fib deposition, exhibit more severely disordered immunologic function, inflammatory reactions and hypercoagulability; glomerular damage in these patients may also be more severe.

## Background

Henoch-Schönlein purpura nephritis (HSPN) is the most common secondary glomerular disease in children. The disease is characterized by glomerular mesangial proliferation and dominant IgA immune complex deposition in mesangial regions and the capillary loops [1, 2]. HSPN is a small-vessel leukocytoclastic vasculitis that exhibits hematuria and proteinuria associated with purpuric rash with or without abdominal pain and/or arthralgia. The reported incidence of HSPN varies from 20 to 80% [3].

Intraglomerular coagulation is an important pathogenetic mechanism leading to glomerular damage. Fibrinogen (Fib), the most abundant coagulation factor, is also involved in cellular interactions, inflammatory responses, and tissue repair in addition to its coagulation function. Previous studies have suggested that Fib/fibrin is involved in various renal diseases in children [4], including HSPN. Recent reports have demonstrated that glomerular Fib deposition plays an important role in the pathogenesis of crescentic HSPN and that decreased Fib deposits predicts histological regression in children with crescentic HSPN [5, 6]. In the present study, we aimed to further evaluate the clinical significance of glomerular Fib deposition by comparing clinical and pathological characteristics among groups with different degrees of Fib deposition.

## Methods

### Patients

Children with HSPN who underwent renal biopsies at the Children’s Hospital of Soochow University from January 2015 to March 2017 were enrolled in this study (age range, 3–18 y). HSPN was diagnosed when hematuria and/or proteinuria were associated with such characteristic manifestations as purpuric skin eruption and abdominal or joint pain. Skin involvement was mandatory for the diagnosis and present in all enrolled patients. Patients with clinical or serological evidence for systemic lupus erythematosus or other vasculitides were excluded. Therapeutic regimens for HSPN patients were decided based on clinical and pathological severities according to the 2012 KDIGO clinical practice guidelines for HSP nephritis.

The patients were divided into different groups according to whether the renal tissue had Fib deposition and the intensity of Fib deposition when present. The clinical, laboratory and pathological characteristics were compared among the groups.

All patients were followed up once every two weeks or month until remission and then every three or six months thereafter to evaluate treatment outcome and adjust drug dosing. Periodical urinalysis and biochemical profiles were performed. The eGFR decline rate was calculated yearly. The treatment outcome was classified as follows [7]: Status A-Normal, the patient was normal on physical examination with normal urine and renal function; Status B-Minor urinary abnormality, the patient was normal on physical examination with microscopic hematuria or proteinuria < 1 g/24 h (or < 40 mg/h/m^2^) or both; Status C-Active renal disease, the patient had proteinuria > 1 g/24 h (> 40 mg/h/m^2^) or hypertension (mean blood pressure > 105 mmHg) or both and GFR > 60 ml/min/1.73 m^2^; Status D-Renal insufficiency: the patient had GFR < 60 ml/min/1.73 m^2^ or died.

### Clinical and laboratory information

The clinical and laboratory findings of the patients at the time of renal biopsy were extracted from the medical records and retrospectively reviewed. Renal biopsy was performed within 2 to 12 weeks of the initial diagnosis of HSPN. Indications for renal biopsy at our institution are as follows [8]: HSP patients present hematuria (gross or microscopic) and/or proteinuria with or without edema, hypertension and renal insufficiency, and no contraindications, especially in those with proteinuria as their first or primary manifestation.

At our institution, cellular immunity and the urine protein spectrum were used as routine tests to evaluate HSP patients’ cellular immunity function and early renal damage based on the literature reports [9, 10]. Cellular immunity tests included the following lymphocyte subsets: CD3+, CD3 + CD4+, CD3 + CD8+, CD4+/CD8+, CD3-CD19+, CD19 + CD23+ and CD3-CD16 + 56+. Components of the urine protein spectrum included N-acetylglucosaminidase (UNAG), α1-microglobulin (Uα1MG), β2-microglobulin (Uβ2MG), IgG (UIgG), microalbumin (UMA) and transferrin (UTRF). Urinary creatinine (Cr) was also measured, and the above proteins were normalized to Cr.

### Clinical classification

HSPN patients were divided into the following seven types based on clinical manifestation according to HSPN clinical classification criteria established by the Pediatrics Nephrology Professional Group of the Chinese Medical Association [8]: isolated hematuria, isolated proteinuria, hematuria and proteinuria, acute nephritis, nephrotic syndrome, rapidly progressive glomerulonephritis and chronic glomerulonephritis.

The criteria of each clinical type are as follows. (1) isolated hematuria: clinical manifestation exclusively involves non-symptomatic hematuria without edema, hypertension or kidney function damage; (2) isolated proteinuria: clinical manifestation exclusively involves non-symptomatic proteinuria without edema, hypertension or kidney function damage; (3) hematuria and proteinuria: clinical manifestation involves both hematuria and proteinuria without edema, hypertension or renal function damage; (4) acute nephritis: acute onset that is similar to acute nephritis; however, few children have the three major symptoms of hematuria, edema and hypertension at the onset; the vast majority of patients only exhibit hematuria and proteinuria at the beginning; (5) nephrotic syndrome: clinical manifestation involves the performance characteristics of a typical nephrotic syndrome, namely, large amounts of proteinuria (24 h protein > 50 mg/kg), hypoproteinemia (serum albumin < 25 g/L), hyperlipidemia (serum cholesterol > 5.7 μmol/L) and edema; (6) rapidly progressive glomerulonephritis: the onset is rapid, oliguria or anuria is noted in the early stage with obvious azotemia, and the condition deteriorates sharply; (7) chronic glomerulonephritis: slow onset, renal damage is persistent after purpura subsides, and the condition is often accompanied by renal dysfunction.

### Pathological grading

The pathological examination included light microscopy, immunofluorescence and electron microscopy. The immunofluorescence examination included evaluation of the deposition of IgA, IgG, IgM, C3, C4, C1q and Fib in the kidney. Immunofluorescence assays were conducted with fresh frozen sections and performed immediately after slicing.

Glomerular changes were graded using the classification of the International Study of Kidney Disease in Children (ISKDC) [7] as grade I (minimal alterations), grade II (mesangial proliferation), grade IIIa (focal) or IIIb (diffuse proliferation or sclerosis with 50% crescents), grade IVa (focal) or IVb (diffuse proliferation or sclerosis with 50–75% crescents), grade Va (focal) or Vb (diffuse proliferation or sclerosis with 75% crescents), or grade VI (membranoproliferative glomerulonephritis).

### Statistical analysis

Statistical analysis was performed using SPSS version 22.0 (IBM Corp., Armonk, New York, USA). Quantitative data were expressed as means ± standard deviations (SDs) or medians and interquartile ranges [M (25th, 75th percentiles)] based on whether the data were normally distributed. Qualitative data were expressed as cases and rates. Multiple constituent ratios were compared using the chi-square test. Analyses of sequential contingency table data were performed using Spearman’s rank correlation test and/or the Kruskal-Wallis rank sum test. Differences among multiple groups were assessed using ANOVA or the Kruskal-Wallis rank sum test followed by pairwise comparisons. A two-sided *P* of < 0.05 was considered statistically significant in all analyses.

## Results

### General characteristics

**Fig. 1:**
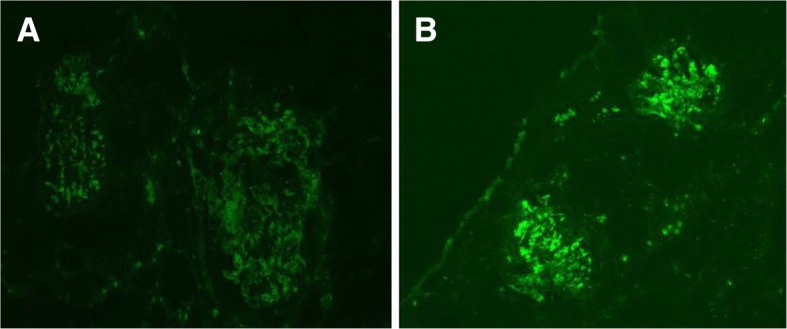
Immunofluorescence staining of fibrinogen in glomeruli (× 200). **a** Fibrinogen deposition in a (+) case; (**b**) Fibrinogen deposition in a (++) case

Eighty-two children with HSPN (53 boys, 29 girls; mean age 8.92 ± 2.49 y) were recruited to our study. Renal biopsies contained adequate numbers of glomeruli for light microscopy and immunofluorescence assessment. The median numbers of glomeruli were 20 for light microscopy and 13 for immunofluorescence. Diffuse, global, granular Fib deposition in the mesangial regions and capillary loops was identified in 64 patients (78.05%), and the intensity of Fib deposition ranged from ± to +++ (Fib±: *n* = 1; Fib+: *n* = 23; Fib++: *n* = 37; Fib+++: n = 3). No Fib deposition was observed in 18 cases (Group 1). Fib deposition in the renal tubules was not observed in any patients. Immunofluorescence staining of glomerular Fib deposition is presented in Fig. 1a-b. Given that Fib± and Fib+++ groups contained fewer cases, these two groups were incorporated into the adjacent groups for follow-up statistical analysis to avoid research conclusion bias: 1 case from the Fib± group was merged into the Fib+ group (Group 2, Fib mild deposition, *n* = 24), and 3 cases from the Fib+++ group were merged into the Fib++ group (Group 3, Fib severe deposition, *n* = 40).

No significant differences in sex ratio and age were noted among the three groups (*P* > 0.05 for both). All patients had normal blood pressure and liver and kidney function. Of the 82 children with HSPN, renal injury developed within 1 month of purpura onset in 63 cases (76.83%), within 1–3 months in 11 (13.41%), within 3–6 months in 6 (7.32%), and within more than 6 months in 2 (2.44%). No statistical difference in the constituent ratio of the occurrence time of kidney involvement was noted among the groups (*P* > 0.05).

### Fib deposition and clinical classification

The clinical classification of 82 patients included 13 cases of isolated hematuria, 14 cases of isolated albuminuria [(21.26 ± 15.72) (1.32~ 44.79) mg/kg/24 h of 24 h U-TP and (13.34 ± 9.74) (0.69~ 28.41) mg/kg/24 h of 24 h U-MA], 50 cases of both hematuria and albuminuria [(29.79 ± 37.97)(1.35~ 199.20) mg/kg/24 h of 24 U-TP and (14.28 ± 13.07) (0.28~ 64.81) mg/kg/24 h of 24 U-MA], 3 cases of acute nephritis and 2 cases of nephrotic syndrome. No correlation between Fib deposition intensity and clinical classification was observed based on both Spearman’s rank correlation test (*r*_s_ = 0.14, *P* = 0.218) and the Kruskal-Wallis rank sum test (*P* > 0.05).

### Fib deposition and laboratory parameters

Statistical differences in the levels of high-sensitivity C-reactive protein (hs-CRP), D-dimer (DD), the proportion of CD19 + CD23+ lymphocyte subsets and UMA/Cr levels were noted among the three groups (Table 1). The hs-CRP level of Group 3 was significantly increased compared with Groups 2 and 1 (*P* < 0.001 for both), and no significant difference was noted between Groups 2 and 1 (*P* = 0.647). The DD levels of Groups 3 and 2 were both significantly increased compared with Group 1 (*P* = 0.019 and *P* = 0.009, respectively), and no significant difference was noted between Groups 3 and 2 (*P* = 1.000). The proportion of CD19 + CD23+ cells in Group 3 was significantly increased compared with Groups 2 and 1 (*P* = 0.007 and *P* = 0.011, respectively). The proportion of CD19 + CD23+ cells in Group 2 was slightly increased compared with Group 1, but the difference was not statistically significant (*P* = 0.858). The UMA/Cr level in Group 3 was increased compared with Groups 2 and 1 (*P* = 0.048 and *P* = 0.003, respectively), but no significant difference was noted between Groups 2 and 1 (*P* = 0.235).

**Table 1:** Comparison of laboratory findings with statistical difference amongthree groups

Parameters	Group1	Group 2	Group 3	*P*
	(*n* = 18)	(*n* = 24)	(*n* = 40)	
hs-CRP (mg/L)	0.30 (0.21~ 2.47)	0.62 (0.54~ 3.14)	7.60 (4.06~ 21.83)	0.000
DD (μg/L)	339.50 (127.50~ 652.00)	666.50 (383.00~ 2725.50)	801.50 (293.75~ 1618.50)	0.007
CD19 + CD23+	6.82 ± 1.79	7.63 ± 3.17	10.33 ± 4.45	0.006
UMA/Cr (mg/g)	208.22 (35.94~ 530.21)	378.20 (163.24~ 609.97)	498.81 (213.88~ 811.18)	0.012

Values are means ± standard deviations or medians and interquartile ranges [M (25th, 75th percentiles)]

*hs-CRP* high-sensitivity C-reactive protein, *DD* D-dimer, *UMA/Cr* urinary microalbumin /urinary creatinine

Moreover, no marked statistical differences in the levels of serum total protein, albumin, cholesterol, triglycerides, IgA, IgG, IgM, C3, C4, Fib or other lymphocyte subsets as well as levels of 24 U-TP/kg, 24 U-MA/kg, UNAG/Cr, Uα_1_MG/Cr, Uβ_2_MG/Cr, UIgG/Cr or UTRF/Cr were noted among the groups (data not presented).

### Fib deposition and pathological grading

No association between Fib deposition intensity and pathological grade (*r*_*s*_ = 0.13, *P* = 0.252) was observed based on Spearman’s rank correlation test, and no marked significant difference was noted between Fib deposition intensity and pathological grade using the Kruskal–Wallis rank sum test (*P* > 0.05; Table 2). No association between Fib deposition intensity and number of crescents was noted in ISKDC class III – IV patients (*r*_*s*_ =  0.02, *P* = 0.910).

**Table 2:** Pathological grades in each group

	Pathological grade (n (%))				Total
	I	II	III	IV	
Group 1	2 (11.11)	10 (55.56)	6 (33.33)	0 (0.00)	18
Group 2	4 (16.67)	11 (45.83)	8 (33.33)	1 (4.17)	24
Group 3	5 (12.50)	15 (37.50)	18 (45.00)	2 (5.00)	40
Total	11 (13.42)	36 (43.90)	32 (39.02)	3 (3.66)	82

### Fib deposition and pathological parameters of immunofluorescence

Glomerular IgA deposition was observed in 76 of the 82 cases (Table 3). A markedly positive correlation was observed between Fib deposition and IgA deposition using the Spearman’s rank-correlation test (*r*_s_ = 0.64, *P* < 0.001), and a statistical difference between Fib deposition and IgA deposition was noted based on the Kruskal–Wallis rank sum test (*H* = 35.47, *P* < 0.001). Renal tubular IgA deposition was detected in 2 cases from Group 1 but was not identified in any cases from Group 2 or 3.

**Table 3:** IgA deposition in each group

	IgA deposition [n (%)]					Total
	–	±	+	++	+++	
Group 1	6 (33.33)	1 (5.56)	1 (5.56)	10 (55.55)	0 (0.00)	18
Group 2	0 (0.00)	0 (0.00)	1 (4.17)	14 (58.33)	9 (37.50)	24
Group 3	0 (0.00)	0 (0.00)	1 (2.50)	9 (22.50)	30 (75.00)	40
Total	6 (7.32)	1 (1.22)	3 (3.66)	33 (40.24)	39 (47.56)	82

A statistical difference in glomerular IgG deposition was noted among the three groups (*P* = 0.027). Pairwise comparisons revealed that the incidence of glomerular IgG deposition in Group 3 was increased compared with Group 1 (*P* = 0.024), but no significant differences were noted between Groups 3 and 2 or between Groups 2 and 1. No marked differences were noted in renal tubular IgG deposition among the groups. No differences in glomerular or renal tubular deposition of IgM, C3 or Cq1 were noted among the groups.

### Fib deposition and the outcome of the patients

Follow-up information was available for all 82 patients with follow-up times ranging from 15 months to 40 months and a median follow-up time of 24 months. No correlation existed between Fib deposition and outcome (*r*_*s*_ =  0.02, *P* = 0.910). The clinical outcome was not significantly different among the three groups (*P* > 0.05, Table 4).

**Table 4:** The clinical outcome of each group

	Clinical outcome [n (%)]				Total
	A	B	C	D	
Group 1	12 (66.67)	6 (33.33)	0 (0.00)	0 (0.00)	18
Group 2	15 (62.50)	9 (37.50)	0 (0.00)	0 (0.00)	24
Group 3	23 (57.50)	17 (42.50)	0 (0.00)	0 (0.00)	40
Total	50 (60.98)	32 (39.02)	0 (0.00)	0 (0.00)	82

## Discussion

In addition to its role in coagulation, the pathogenesis of crescents, glomerular sclerosis and reduced renal functioning [5, 6, 11, 12], Fib exhibits an increasing number of additional functions. Fib and its derivatives have proinflammatory properties and can induce immune cell activation [13, 14]. Fib may be toxic to podocytes, and Fib deposition in the damaged podocytes of a proteinuric mouse model of FSGS accelerates podocyte damage and promotes the progression of glomerular injury by inducing the expression of inflammatory cytokines [15]. Moreover, the presence of Fib increases the permeability of the glomerular capillary wall and non-specific trapping within the glomerulus [16]. Recent studies have demonstrated that Fib and its degradation products act as strong mitogens and play a major role in renal interstitial fibrosis in kidney disease by stimulating renal fibroblast proliferation in a dose-dependent manner through TLR2-, TLR4-, and ICAM-1-dependent signaling in unilateral ureteral occlusion (UUO). In addition, Fib depletion caused by gene modification or drug treatment offers vital protection against tubulointerstitial damage and disruption caused by UUO and folic acid nephropathy [17, 18].

Our study demonstrated that 76.83% of children with HSPN exhibited Fib deposition in the glomerular mesangial area and capillary loops. Similarly, in a prospective comparative study of 137 children with HSPN and 41 children with IgAN, 97 (70.80%) cases with HSPN exhibited Fib deposition [19]. Although Fib deposition did not correlate with clinical classification, it is necessary to recruit more HSPN patients with clinical manifestation of nephritis and nephropathy to further explore the relevance of Fib deposition given the rarity of cases with acute nephritis or nephrotic syndrome. In our study, Fib deposition was not present in six patients with pathological grade III, although clinical and experimental observations have indicated that Fib/fibrin play important roles in the pathogenesis of crescents. Studies have confirmed that crescents are cellular at the onset of the disease and evolve with time towards a fibrous phenotype. In addition, the composition of crescents may exhibit marked differences within days in patients with active disease. The interval between disease onset and the time of renal biopsy may impact histopathological findings. All crescents observed in the six patients were cellular, and renal biopsies were performed in the early stage of renal involvement onset, potentially explaining why no Fib deposition was observed. Given that the majority of crescents in all patients are cellular and a small portion of crescents are fibrocellular crescents, many patients in our study lacked fibrous crescents. Thus, no correlation between Fib deposition intensity and the number of crescents in ISKDC class III – IV patients was observed.

Our study found that hs-CRP levels in patients with severe Fib deposition were increased compared with patients with mild or no Fib deposition. Fib and fibrin may promote inflammation by inducing synthesis of proinflammatory cytokines, such as IL-1 β, IL-6 and TNF-α, from peripheral blood mononuclear cells [20, 21]. Thus, we surmise that increased Fib induces hs-CRP production in children with HSPN. Our results demonstrated that DD levels in the settings of both severe and mild Fib deposition were significantly increased compared with patients with no Fib deposition, suggesting that patients with Fib deposition exhibit increased blood hypercoagulability and are more likely to develop renal microthromboses. The immune function disorder in HSP is characterized by the dominant activation of type 2 helper T lymphocytes and polyclonal B lymphocytes [22]. Levels of activated B cells (CD19 + CD23+ cells) in patients with severe Fib deposition were increased compared with patients with either mild Fib deposition or no Fib deposition in our study. These results suggest that the degree of Fib deposition is associated with the number of activated B lymphocytes, and patients with severe Fib deposition exhibit markedly increased numbers of activated B lymphocytes.

In our study, patients with severe Fib deposition exhibited increased UMA/Cr levels compared with patients with either mild or no Fib deposition. Given that differences in the other indices in the urine protein profile were not statistically significant among the three groups, this study suggests that patients with severe Fib deposition may have serious glomerular lesions. Fib acts on endothelial cells directly via its C-terminal end. Alternatively, Fib can combine with the intercellular adhesion molecule 1 receptor and activate the extracellularly regulated protein kinase signaling pathway by affecting the expression of tight junction proteins 1 and 2, causing increased microvascular permeability and endothelial cell dysfunction and leading to liquid and protein leakage [16, 23]. Therefore, the level of proteinuria in patients with Fib deposition is hypothesized to be increased compared with patients without Fib deposition. Our finding of significantly increased urinary microalbumin levels in children with severe Fib deposition supports the aforementioned theory. However, no differences in the 24 h total urinary protein or albumin levels were observed among the three groups. Thus, the action of Fib on endothelial cells and the correlation between Fib deposition and urinary protein should be further explored in children with HSPN.

Although Fib deposition was not related to pathologic grade, Fib deposition had a markedly positive correlation with IgA deposition. Moreover, HSPN children with high Fib deposition exhibit an increased incidence of glomerular IgG deposition. Renal IgG deposition is associated with a worse overall renal outcome, and a trend toward a worse outcome with renal Fib deposition has been reported [24]. These results suggest that Fib deposition may play an important role in immunoglobulin deposition and renal tissue injury. It is possible that three phenomena—Fib deposition, immune complex deposition and renal injury—form a vicious cycle. Our research supports this opinion.

In our subjects, 6/82 patients with HSPN did not exhibit glomerular IgA deposition. Uncommon renal pathological manifestations other than predominant mesangial IgA depositions have been reported in patients with HSPN [25, 26]. Tanaka H et al. reported one case of HSPN without IgA deposits and proposed that non-lgA mesangial proliferative glomerulonephritis should be considered a cause of urinary abnormalities in HSPN [25]. West CD et al. [26] reported that the early renal biopsy findings of ten patients with HSPN revealed segmental endocapillary proliferative glomerulonephritis without predominant mesangial IgA deposits. In the second biopsies, 3 patients revealed IgA deposition with a diffuse mesangial distribution typical of that commonly observed in HSPN. Because these 6 patients did not exhibit urinary abnormalities before the development of the purpura rash, their renal involvement was diagnosed as glomerulonephritis associated with HSP.

The study failed to demonstrate a relationship between Fib deposition and the clinical outcome. The initial renal biopsy may not forecast the clinical outcome of HSPN because patients with mild histopathological disease activity are typically administered less aggressive immunosuppressive therapy, which importantly influences the outcome of HSPN [27]. Similarly, our study demonstrates that Fib deposition severity in the initial renal biopsy is not necessarily a reliable prognostic factor. One possible reason is the aggressive treatment provided to patients with severe Fib deposition. Moreover, regarding long-term follow-up, our follow-up time is relatively short. Therefore, serial biopsies and prolonged follow-up time might aid in ascertaining the ultimate outcome of HSPN patients with Fib deposition.

There are two limitations to this study. First, the study was retrospective, so causality could not be determined. Second, the time of follow-up is relatively short, which may affect the statistical results of the correlation between Fib deposition and clinical outcomes.

## Conclusion

To our knowledge, this is the first study that compared clinical and pathological differences among patients grouped based on Fib deposition levels. The data indicate that HSPN children with glomerular Fib deposition, especially those with severe Fib deposition, have more severe inflammatory reactions, hypercoagulability and disordered immune functions; glomerular damage in these patients may also be more severe.

## Abbreviations

24 h U-MA: 24-h Urinary microalbumin
24 h U-TP: 24 h Urinary total protein
DD: D-dimer
Fib: Fibrinogen
hs-CRP: high-sensitivity C-reactive protein
HSPN: Henoch-Schönlein purpura nephritis
UIgG/Cr: Urinary immunoglobulin G/urinary creatinine
UMA/Cr: Urinary microalbumin /urinary creatinine
UNAG/Cr: Urinary N-acetylglucosaminidase/urinary creatinine
UTRF/Cr: Urinary transferrin / urinary creatinine
Uα1MG/Cr: Urinary α1 microglobulin/urinary creatinine
Uβ2MG/Cr: Urinary β2 microglobulin/urinary creatinine
